# Amino Acid Transporters in Plant Cells: A Brief Review

**DOI:** 10.3390/plants9080967

**Published:** 2020-07-30

**Authors:** Guangzhe Yang, Qiuxing Wei, Hao Huang, Jixing Xia

**Affiliations:** State Key Laboratory of Conservation and Utilization of Subtropical Agro-bioresources, College of Life Science and Technology, Guangxi University, Nanning 530005, China; weiqiux@163.com (Q.W.); 1908301017@st.gxu.edu.cn (H.H.)

**Keywords:** amino acids, transporter, uptake, phloem loading, phloem unloading, xylem-phloem transfer, development regulation, stress tolerance, defense response, nitrogen use efficiency

## Abstract

Amino acids are not only a nitrogen source that can be directly absorbed by plants, but also the major transport form of organic nitrogen in plants. A large number of amino acid transporters have been identified in different plant species. Despite belonging to different families, these amino acid transporters usually exhibit some general features, such as broad expression pattern and substrate selectivity. This review mainly focuses on transporters involved in amino acid uptake, phloem loading and unloading, xylem-phloem transfer, import into seed and intracellular transport in plants. We summarize the other physiological roles mediated by amino acid transporters, including development regulation, abiotic stress tolerance and defense response. Finally, we discuss the potential applications of amino acid transporters for crop genetic improvement.

## 1. Introduction

Nitrogen (N) is an essential macroelement for plant growth and development. Plants mainly absorb inorganic N in the form of nitrate and ammonium from the soil, while organic N such as amino acids, can also be taken up by plants [1]. The absorbed inorganic N can be assimilated into amino acids in roots and/or leaves. Amino acids in roots are transported mainly to source leaves in the xylem transpiration stream [2]. As the main transport form of organic N in most plant species, amino acids synthesized in leaves or derived from roots are transported via phloem to developing sink organs to meet their N requirement [3]. Therefore, the transport and allocation of amino acids within plants is crucial for their growth, development and seed set.

Amino acids transport is generally mediated by transport proteins in plants. The first plant amino acid transporters were cloned mainly through functional complementation of yeast mutants deficient in amino acid transport [3,4,5]. Subsequently, amino acid transporters could be identified by bioinformatics analysis after the completion of genome sequencing of some plants [6,7]. Now, the presence of dozens, or even hundreds, of amino acid transporters are found in different plant species (Table 1). These transporters belong to three major families: ATF (amino acid transporter family, also called AAAP family), APC (amino acid-polyamine-choline transporter family) and the newly identified UMAMIT (usually multiple acids move in and out transporter family) [8]. The ATF family generally contains eight subfamilies: AAP (amino acid permeases), LHT (lysine and histidine transporters), ProT (proline transporters), GAT (γ-aminobutyric acid transporters), ANT (aromatic and neutral amino acid transporters), AUX (auxin transporters), ATL (amino acid transporter-like) and VAAT (vesicular aminergic-associated transporters) [9]. The members of the AUX subfamily usually transport auxin instead of amino acids [8,10]. The APC family consists of three subfamilies: CAT (cationic amino acid transporters), ACT (amino acid/choline transporters) and PHS (polyamine H^+^-symporters) [11]. UMAMITs belong to the nodulin-like gene family [12], and recently several members of this family have been reported to function as bidirectional amino acid transporters [13,14]. Among these families, of main attention in plants is the AAP family. With respect to the phylogenetic relationship of these transporters, readers are referred to other literature [7,15,16]. Despite belonging to different families, these amino acid transporters display some common properties such as broad expression pattern and substrate specificity. Here, we focus mainly on the amino acid transport processes and the other physiological roles mediated by these transporters. The potential applications of amino acid transporters for crop genetic improvement are also discussed in this review.

## 2. General Features of Plant Amino Acid Transporters

### 2.1. Broad Expression Pattern

Determining the gene expression pattern is crucial for studying the function of plant amino acid transporters. Several technologies such as northern blot, reverse transcription-polymerase chain reaction (RT-PCR) and the transcriptional GUS (β-glucuronidase) reporter assay have been employed to detect the expression pattern of individual amino acid transporters. Recently, high throughput analyses such as RNA sequencing have been used to simultaneously detect the expression of multiple amino acid transporters [9,18,22]. Although the expression levels of individual amino acid transporter may differ, most of them are ubiquitously expressed throughout the plant with relatively high abundance in certain organs [26,27,28,29]. For example, *StAAP1* exhibits high expression in mature leaves of potato, but weak expression in roots, stems, sink tubers and sink leaves [26]. The transcript of *AtANT1* was detected in all Arabidopsis organs with highest abundance in flowers and cauline leaves [27]. In addition, the transcript levels of some amino acid transporters change at different development stages or under certain stress conditions, suggesting that their expression is developmentally or environmentally regulated [19,22,24]. Overall, the broad expression patterns imply that each amino acid transporter may process multiple physiological functions in planta. Of note, the well-studied *AAP* family genes are found to be usually expressed in the vascular system of plants [29,30,31], suggesting that they play important roles in the long-distance transport of amino acids.

**Figure 1:**
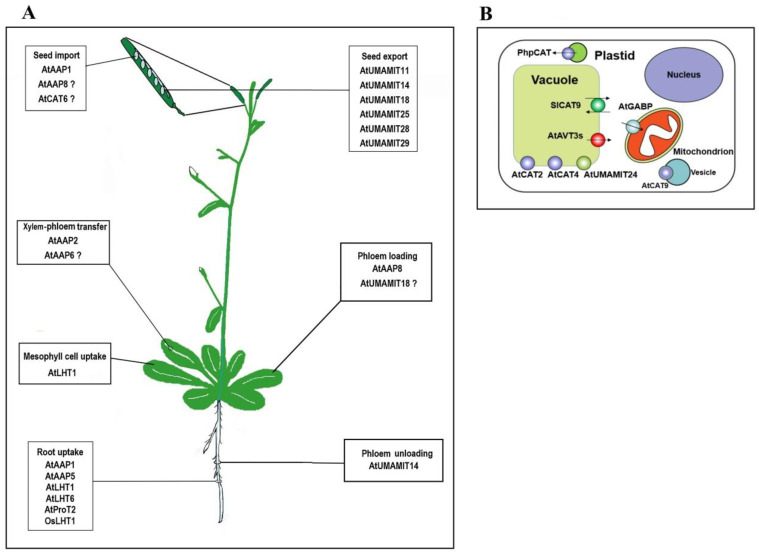
Overview of the function of amino acid transporters identified in plants. (**A**) Summary of transporters involved in intercellular and long-distance transport of amino acids. Some genes that were proposed to participate in certain amino acid transport steps are marked with question marks due to lack of direct evidence. (**B**) Summary of transporters involved in intracellular transport of amino acids. Transporters with defined transport direction are marked with black arrows. References on these genes depicted in the figure can be found in the main text.

With respect to their subcellular localization, most of the reported plant amino acid transporters are located at the plasma membrane, while only a few of them are localized to the organelle membrane (Figure 1B). For instance, the Arabidopsis GABA (γ-aminobutyric acid) transporter AtGABP (also called BAT1 [32]), and cationic amino acid transporter AtCAT9 are localized on the mitochondrial and vesicular membranes, respectively [33,34]. These organelle-localized transporters mediate the intracellular translocation of amino acids between different compartments, which is important for their synthesis, conversion and storage [8,35]. Nevertheless, very few studies have been reported on such transporters up to now, which will get more attention in future studies.

### 2.2. Broad Substrate Selectivity

The substrate specificity of plant amino acid transporters has been extensively studied in planta [36] and in heterologous systems such as yeast [37,38] and *Xenopus laevis* oocytes [39,40]. Most of them are proton-amino acid symporters [3,4,41]. With the exception of the reported ProT and GAT family members [28,42,43], the other family members, including AAPs, LHTs, CATs and ANTs, are usually capable of transporting a broad spectrum of amino acids with preference for some amino acids [3,27,37,44,45]. For example, OsAAP3 can mediate the transport of neutral, acidic and basic amino acids, but transports the basic amino acids lysine and arginine relatively well [40]. AtLHT2 can recognize amino acids with different charges but transports neutral amino acids with high efficiency [38]. The ProT members, including AtProT1, AtProT2 and LeProT1, seem to selectively transport proline compared with the other proteinogenic amino acids [28,42]. This substrate preference might be related to the demand for proline during plant development, since some *ProTs* such as *LeProT1* are specifically expressed in flowers (especially in pollen) that accumulate much more proline compared with vegetative tissues [42]. Very few GAT members have been reported so far, and AtGAT1 from Arabidopsis is described as a highly selective, high-affinity GABA transporter [43].

Besides amino acids, other substrates can also be transported by some amino acid transporters. These substrates are mainly amino acid-related compounds, including indole-3-acetic acid (IAA) [27], glycine betaine [42,46], choline [42], 1-aminocyclopropane-1-carboxylic acid (ACC) [47,48], amino acid-based pesticides [49,50,51] and so forth. For example, two members of LHT family, AtLHT1 and AtLHT2, have been reported to be able to transport the biosynthetic precursor of ethylene, ACC, which is also a non-proteinogenic α-amino acid [47,48]. Disruption of *AtLHT1* impairs the exogenous ACC-induced ethylene responses, while overexpression of *AtLHT1* or *AtLHT2* can restore the ACC-resistance phenotype of *atlht1* mutants [47,48]. More recently, several studies have shown that AtLHT1 can also mediate the translocation of amino acid-pesticide conjugates within plants [49,50]. Although ProT proteins fail to transport efficiently proteinogenic amino acids other than proline, they exhibit higher affinity for glycine betaine than for proline [42,46], so they may be involved in the transport of these compatible solutes in plants. The multiplicity of substrates of some amino acid transporters also suggests that they may play various physiological roles in planta.

### 2.3. Multiple Physiological Functions of Some Amino Acid Transporters

The in planta functions of amino acid transporters have been extensively studied with genetic, physiological and biochemical methods in the past two decades. They participate in multiple amino acid transport processes within plants, including amino acid intracellular and intercellular translocation, uptake from medium, phloem loading and unloading, xylem-phloem transfer and post-phloem delivery into sink cells (Figure 1; see below). It is worth noting that several amino acid transporters have been reported to have more than one physiological role in planta [14,37,52,53,54]. For example, initial studies demonstrated a function of AtAAP1 in root amino acid uptake from medium [52]. Subsequently, Sanders et al. [53] found that AtAAP1 also mediates the import of amino acids into developing embryos. AtLHT1 was shown to participate in amino acid import in roots as well as in mesophyll cells, so its knockout mutants displayed retarded growth when grown on aspartate or glutamate as the sole N source, and decreased amino acid uptake in mesophyll protoplasts [37]. Similarly, AtUMAMIT14 is involved in phloem unloading of amino acids in root and seed [14,54]. The pleiotropy of these transporters poses challenges in studying their function and interpreting obtained results. Thus, it is necessary to keep this pleiotropy in mind when performing experiments to dissect their functions. For example, to exclude the possible interference of AtAAP1 function in root amino acid uptake with its role in the seed, the researchers only supplied amino acid-free N fertilizer to plants in the study [53]. On the other hand, these amino acid transporters may have overlapping, at least partially overlapping, functions within plants, as indicated by no visible phenotypic changes observed in some of their knockout mutants [55,56,57].

## 3. Amino Acid Transport Processes Mediated by Amino Acid Transporters

### 3.1. Acquisition of Amino Acids by Roots

Several amino acid transporters, including AtLHT1 [37], AtLHT6 [58], OsLHT1 [59], AtAAP1 [52], AtAAP5 [60] and AtProT2 [61], have been reported to be able to take up amino acids from external medium (Figure 1A). These findings are mainly based on the observations that mutants defective in certain amino acid transporters show growth retardation on medium containing amino acid as the sole N source [37], reduced uptake of amino acids [36,58] or better growth (survival) on toxic levels of some amino acids or their analogues [52,58]. The uptake characteristics of these transporters generally differ in amino acid type and concentrations. AtLHT1 is involved in uptake of glutamine, alanine, glutamate and aspartate at low concentrations, but not arginine or lysine, while AtAAP5 can mediate the uptake of arginine and lysine under the same condition [36]. Unlike AtLHT1 and AtAAP5, AtAAP1 may function in amino acid acquisition only at high concentrations, as its mutation does not change the uptake of any of the tested amino acids at low concentrations [36].

It is noteworthy that experiments determining the uptake function of plant amino acid transporters are generally performed in agar medium or nutrient solutions [36,37,52,58] which contain enough free amino acids and are usually under appropriate pH and sterile conditions. However, the soil environment for growing crops is much more complex than these simulated environments, such as having very low concentrations of free amino acids [62,63], inappropriate (neutral or alkaline) pH or strong competition for amino acids with large amounts of soil microorganisms [64,65]. Therefore, it remains to be determined whether these transporters are capable of taking up amino acids from soils, and how much they contribute to plant N acquisition in a cropland system.

### 3.2. Phloem Loading

The growth and development of sink organs depends on amino acids supplied by source leaves, and their phloem loading is a bottleneck in the source-to-sink translocation of N. The amino acids synthesized in mesophyll cells can be loaded into phloem via a symplastic or apoplastic transport path, depending on the presence or absence of plasmodesmata between phloem parenchyma and companion cells, and their frequency [66]. Arabidopsis and most crops are suggested to be apoplastic phloem loaders [2], in which phloem loading of amino acid requires two key steps. Amino acids are firstly exported out of the phloem parenchyma cells of the minor veins into the apoplasm via passive transport, and then are pumped into the phloem via active transport [2,67]. In this pathway, the bidirectional amino acid transporter SiAR1/AtUMAMIT18 is considered to mediate amino acid export into the apoplasm [66,67] as it is expressed in both the major and minor veins of source leaves [13]. However, Besnard et al. [54] found that loss-of-function of AtUMAMIT18 does not change amino acid composition in leaf phloem exudates. Hence, further research is needed to determine whether AtUMAMIT18 plays a role in amino acid export from phloem parenchyma cells, or which facilitator compensates for the loss of AtUMAMIT18 function.

Several amino acid importers such as AtAAP2 [30], AtAAP8 [68] and AtProT1 [46] have been shown to be expressed in the leaf phloem, but only AtAAP8 is determined to function in amino acid phloem loading from leaf cells so far [68]. When ^14^C-labeled glutamate or glutamine was fed to source leaves, the label transported to sink leaves and siliques decreased significantly in *ataap8* mutants. In addition, the total amino acid concentrations were also markedly reduced in the *ataap8* leaf exudates [68]. Consequently, decreased phloem loading and source-to-sink transport of amino acids resulted in reduction of silique and seed numbers, and yield in *ataap8* mutants [68]. However, since the phloem loading and source-to-sink transport of amino acids are not completely aborted in *ataap8* plants, other unidentified transporters with overlapping function with AtAAP8 may exist in Arabidopsis, which needs to be further explored in future studies.

### 3.3. Xylem-Phloem Transfer

Root-derived amino acids are transported to the shoot with the xylem transpiration stream. Along the long-distance translocation pathway, some amino acids may move from the xylem to the phloem for direct N supply to sink tissues [2]. This transfer process requires several steps: first, amino acids move from tracheary elements into xylem parenchyma cells, and then move to phloem parenchyma cells via plasmodesmata. Due to the lack of plasmodesmata between phloem parenchyma and companion cells, the amino acids are further exported into the apoplasm followed by import into the sieve element-companion cell (SE-CC) complexes [66]. AtAAP2 and AtAAP6 are thought to function in xylem-phloem transfer of amino acids [30,31]. Owing to the low amino acid concentrations in xylem sap, high-affinity transporters are required to mediate the import of amino acids into xylem parenchyma cells. AtAAP6 can transport acidic and neutral amino acids with low *K*_m_ values [39], and is localized to the xylem parenchyma cells, so it is proposed to function in the import of amino acids diffusing out of the tracheary elements into xylem parenchyma [31]. However, subsequent studies showed that loss of function of AtAAP6 results in marked reduction of total amino acid concentrations in sieve element sap [69]. This result cannot be caused by the reduction in the xylem-phloem transfer of amino acids, as concentrations of amino acids in the xylem are much lower than in the phloem sap [70,71]. Even if the xylem-phloem transfer of amino acids is entirely aborted, the levels of amino acids in phloem sap does not significantly decline. Therefore, additional evidence is required to determine the role of AtAAP6 in xylem-phloem transfer of amino acids. Alternatively, AtAAP6 may have other unknown roles in amino acid transport, which can regulate phloem amino acid concentration in Arabidopsis. 

Unlike AtAAP6, unambiguous evidence supported that AtAAP2 functions in xylem-phloem transfer of amino acids: (a) AtAAP2 is localized in the phloem and particularly in companion cells [30]; (b) total free amino acid levels in the xylem sap of *ataap2* mutants are slightly higher than those in wild type [30]; (c) when roots were fed with ^14^C-labeled glutamine, *ataap2* mutants display a significant increase of ^14^C-label in source leaves versus the wild-type, but a decrease in sink leaves and siliques [30]. These results indicate that knockout of *AtAAP2* leads to reduction in xylem-phloem transfer of amino acids, thereby increasing the transport of root-derived amino acids to mature leaves, while reducing their transport to sinks via phloem. Unexpectedly, enhanced amino acids allocation to leaves has positive effects on *ataap2* growth, yield and nitrogen use efficiency (see below).

### 3.4. Phloem Unloading

Depending on plant species and sink organs, phloem unloading of assimilates may follow a symplastic or apoplastic path. In roots, sink leaves and seeds the unloading mechanism of amino acids is generally thought to be symplastic [10,67], so the exporter responsible for amino acid phloem unloading has not been found for a long time. Recently, the functional characterization of UMAMIT efflux systems has changed the understanding of phloem unloading of amino acids in these organs. Some UMAMITs can facilitate import and export of amino acids with broad substrate specificity [13,14]. In seeds, AtUMAMIT11, AtUMAMIT14 and AtUMAMIT18 are localized in the nutrient-unloading domain, so they may mediate amino acid release from the phloem at the end of the funiculus vasculature [13,14]. AtUMAMIT14 is also expressed in root pericycle and phloem, and its knockout mutants exhibit decreased shoot-to-root and root-to-medium transport of leaf-fed amino acids, so AtUMAMIT14 is suggested to function in phloem unloading of amino acids in root as well [54]. 

### 3.5. Post Phloem Transport

Amino acids released from phloem move to terminal sink cells via symplastic and apoplastic routes, depending on plant species, sink tissues and developmental phase [67]. In roots and sink leaves, post-phloem translocation of amino acids occurs symplastically [67]. In endospermic seeds, however, the three constituting tissues (seed coat, endosperm and embryo) are symplastically isolated from each other, so amino acids need to be exported and subsequently reimported at least two times in these seeds for final uptake by the embryo [66,67]. Consistent with these complex transport pathways, many amino acid importers and exporters have been found to be expressed in seed (Figure 1A). The importers include AtAAP1 [53], AtAAP8 [31] and AtCAT6 [45]. AtAAP1 is expressed in the storage parenchyma and outer epidermis cells of the developing embryo [53]. The total carbon and N amounts are significantly decreased in *ataap1* seeds, whereas the content of total free amino acids are elevated in *ataap1* seed coat/endosperm, suggesting that knockout of *AtAAP1* reduces the uptake of amino acids by the embryo, thereby leading to an accumulation of free amino acids outside the embryo [53]. AtAAP8 is expressed in young seeds [31], and its deletion leads to strong reduction in seed number [72]. Thus, AtAAP8 may be involved in import of amino acids into developing seeds. Except for glutamine, however, no difference in individual amino acid content was detected between *ataap8* and wild-type seeds [72]. Therefore, the exact role of AtAAP8 in amino acid transfer in the seed remains to be resolved. Recently, AtAAP8 was found to function in phloem loading of amino acids in source leaves [68], so the seed phenotype of *ataap8* mutants might also be caused by reduction of source-to-sink transfer of amino acids. Similarly, despite expression in the developing seed, knockout of *AtCAT6* did not change amino acid content in the seed, so the function of AtCAT6 in the seed requires further investigation [45].

Several UMAMIT members expressed in the seed fulfill the function of amino acid export (Figure 1A). These *UMAMIT* genes are expressed in distinct tissues within developing seeds and play different roles in amino acid transfer from the mother to daughter tissues (for a review see [8,10]). For example, AtUMAMIT29 is localized in the middle layer of the inner integument and it may mediate amino acid export from the outer integument to the inner integument [14]. Repression of its expression has no impact on seed set, but seed volume is significantly decreased [14]. The tonoplast-localized AtUMAMIT24 was found mainly in the chalazal seed coat and may be involved in temporary storage of amino acids in chalaza [73]. AtUMAMIT25 is targeted to endosperm cells, indicating its role in amino acid export from the endosperm [73].

### 3.6. Intracellular Translocation of Amino Acids

Amino acid metabolism is compartmented, so their transport between different organelles and cytoplasm occurs frequently. While numerous plasma membrane-localized amino acid transporters have been reported, very few organelle-localized amino acid transporters have been functionally identified so far (Figure 1B). The *Petunia hybrida* cationic amino acid transporter, PhpCAT, is one of the well-studied transporters localized to organelles [74]. Phenylalanine (Phe) is mainly synthesized in plastids, while the synthesis of Phe-derived compounds is a complex multi-compartmental process. Hence, Phe must be first exported to the cytosol across the plastid membrane. PhpCAT, localized to the plastid, was shown to be responsible for plastidial Phe export [74]. Repression of *PhpCAT* expression reduces Phe, tyrosine and tryptophan (to a lesser extent) levels in cytosol, as well as the total emission of Phe-derived volatiles. In contrast, overexpression of *PhpCAT* results in increased levels of Phe-derived volatiles and aromatic amino acid pools in the cytosol [74]. GABA is synthesized in cytosol but is catabolized to other metabolites in mitochondria. AtGABP is suggested to function as a mitochondrial GABA transporter mediating the import of GABA from the cytosol into mitochondria [33]. 

The vacuole can serve as a large amino acid pool in plant cells [75]. Amino acids import into and export out of the vacuole requires the activity of transporters. Several amino acid transporters belonging to different families have been found to be localized on the tonoplast [56,73,76]. AtAVT3 family members are homologs of AtANT1 but localized to the vacuolar membrane in Arabidopsis [76]. When expressed in a yeast mutant defective in amino acid export from vacuoles, they can reduce the accumulation of amino acids within the vacuoles. Thus, AtAVT3 family members are proposed to function as vacuolar amino acid exporters in Arabidopsis [76]. Some CAT family members such as AtCAT2 and AtCAT4 were also found to be localized to the tonoplast [56,77]. Knockout of *AtCAT2* significantly increases the total soluble amino acid content in adult leaves, suggesting the involvement of AtCAT2 in regulation of leaf amino acid levels [56]. Nevertheless, it is unclear that the increase in amino acids is caused by their accumulation in cytosol or vacuoles, so the transport direction of AtCAT2 remains uncertain. 

## 4. Other Physiological Roles of Plant Amino Acid Transporters

### 4.1. Regulation of Plant Development

Altered expression of amino acid transporters can influence internal amino acid homeostasis in plants, ultimately leading to changes in plant metabolism, growth and development. However, these changes may be caused by different factors: first, alterations in plant growth and development may be due to the insufficiency of amino acids as a N source for sinks, which has been outlined in Section 3. On the other hand, besides serving as fundamental nutrients, amino acids can also act as regulators to modulate plant growth and development, which will be summarized in this section.

High concentrations of amino acids inhibit growth in both monocots and dicots [29,52,57,78]. In rice, low levels of amino acids can promote tiller bud outgrowth and tiller formation, while their excessive accumulation retards tiller bud outgrowth and reduces tiller number [29,78]. Altered expression of some amino acid transporters can regulate rice tillering by changing internal amino acid homeostasis [29,78]. The rice amino acid permease 5, *OsAAP5*, is expressed in various organs and may mediate the transport of basic and neutral amino acids in plants. Its expression level differs between indica and japonica rice varieties. Indica cultivars with low expression of *OsAAP5* produce more tillers than japonica cultivars, and vice versa [29]. Reduced expression of *OsAAP5* in japonica varieties significantly reduces the concentrations of some basic and neutral amino acids in the tiller basal part, leaf sheath and leaf blade, whereas tiller number increases. Correspondingly, the opposite results are observed in *OsAAP5*-overexpressing plants [29]. Hormones play important roles in regulating plant development. The levels of cytokinins (CKs) are changed in the modified plants, so *OsAAP5* regulates rice tiller formation probably by affecting CK levels in plant cells [29]. Nevertheless, it is still obscure how amino acid accumulation reduces CK content in rice. Additionally, manipulation of another *OsAAPs* gene, *OsAAP3*, produces a similar effect on rice development as *OsAAP5* [78]. Unlike these two *OsAAP* genes, altered expression of another type of amino acid transporter, *OsLHT1*, has the opposite effect on rice tillering. Knockout of *OsLHT1* reduces rice tiller number and shoot biomass [59,79], suggesting that OsLHT1 and OsAAP3/OsAAP5 might play different roles in transport and allocation of amino acids in rice.

In Arabidopsis, overexpression of *AtCAT1* leads to shoot growth inhibition, earlier flowering and senescence [57]. However, only minor differences in the amino acid profile were found in adult leaves of *AtCAT1*-OE and wild-type plants, whereas the expression of genes involved in salicylic acid (SA) biosynthesis and SA levels are elevated in the transgenic plants, indicating the involvement of SA in the developmental alterations of *AtCAT1*-OE plants [57]. From these examples, it can be assumed that altered expression of amino acid transporters regulates plant development, probably by affecting hormone action.

### 4.2. Abiotic Stress Tolerance

Proline is an important compatible solute in plants, and its accumulation can protect plants from abiotic stresses such as salinity, drought and freezing. Besides enhanced synthesis and decreased degradation, transport of proline within plants may also contribute to its accumulation [80,81] as exemplified by the observation that the proline concentration is sharply increased in the phloem sap of water-stressed alfalfa [82]. Several different types of amino acid transporters, including ProTs, AAPs and LHTs, have been reported to have the activity of transporting proline [37,39,46], and altered expression of some of these transporters can enhance plant stress tolerance [83,84]. In soybean, two ProT genes, *GmProT1* and *GmProT2*, are strongly induced by salt, drought and ABA treatment. Arabidopsis plants over-expressing *GmProT1* or *GmProT2* accumulate more proline relative to wild-type under salt and drought stresses and enhance the expression of stress-related genes. As a result, the overexpressor lines show improved salt and drought tolerance [83]. GABA, another stress-induced compound in plants, can function as an endogenous signaling molecule that regulates plant growth, development and stress responses [85]. When the GABA transporter *PeuGAT3* was overexpressed in Arabidopsis and poplar, the transgenic plants showed increased thickness of xylem cells walls. Furthermore, the roots of transgenic Arabidopsis grow much better than the wild type under salt and drought stresses, suggesting that overexpression of *PeuGAT3* enhances abiotic stress tolerance in Arabidopsis [86].

### 4.3. Defense Response

Plants are frequently infected by diverse pathogens and pests in the natural environment. Upon infection, invaders acquire nutrition from their hosts and amino acids are an important source of N provided by host plants [87]. Therefore, regulating amino acid homeostasis through changing the expression of transporters might influence the plant defense response. AtLHT1 participates in the uptake of amino acids into roots and mesophyll cells [37]. Liu et al. [88] demonstrated that the expression of *AtLHT1* is induced by pathogen infection. Its knockout mutation increases resistance to a broad spectrum of pathogens in an SA-dependent fashion, evidenced by the observation that the induction of *AtLHT1* by pathogen infection and *atlht1*-conferred resistance is diminished in mutants defective in SA pathway. The enhanced disease resistance in *atlht1*may be due to alteration in cellular redox homeostasis and deficiency of cytosolic glutamine [88]. Unlike *AtLHT1*, overexpression of *AtCAT1* enhances resistance of Arabidopsis to bacterial pathogens [57]. As in the *atlht1* mutant, the expression of the SA-synthesis genes *ICS* and endogenous SA amount are significantly elevated in *AtCAT1-*OE plants, suggesting that increased pathogen resistance of the modified plants may also depend on the SA pathway [57]. Nevertheless, the exact link between the altered expression of amino acid transporter and the accumulation of SA is still obscure.

Altered expression of amino acid transporters can also influence the resistance to plant-parasitic pests, such as root-knot and cyst nematodes [89,90,91]. For example, knockout of *AtAAP3* and *AtAAP6* significantly diminished the root-knot nematode infestation levels in Arabidopsis. Both *ataap3* and *ataap6* mutants produced fewer female nematodes, but more males in comparison to wild-type plants. Moreover, nematodes isolated from *ataap3* and *ataap6* mutants exhibit reduced egg hatching, infectivity and lipid energy reserves [89]. Similarly, loss of function of *AtAAP1*, *AtAAP2* or *AtAAP8* markedly reduced the number of female cyst nematodes propagated on these mutant plants [90].

## 5. Potential Applications of Amino Acid Transporters for Crop Improvement

### 5.1. Promoting Plant Growth and Increasing Yields

As the dominant transport form of organic N in plants, the transport and allocation of amino acids is crucial for plant growth and development. Altered expression of amino acid transporters could affect not only plant N metabolism, but also carbon (C) metabolism due to their strong interaction [92,93]. Dependent on the transporters, their overexpression (OE) or knockout may improve plant growth and seed yield. For example, additional copies of *PsAAP1* were overexpressed in pea under control of the *AtAAP1* promoter, which targets expression in the phloem and the cotyledon transfer cells [94]. In *PsAAP1*-OE plants, phloem loading and embryo loading of amino acids, as well as root N uptake and assimilation, and root-to-shoot amino acid delivery are increased. As a result, the *PsAAP1* overexpressors show increased shoot biomass and pod and seed number. Ultimately, the seed yield per plant can be enhanced by up to 33% in the transgenic plants [94]. Moreover, the total seed protein amount per plant is also elevated [94].

Different from *PsAAP1*, down-regulation of some amino acid transporters has positive influence on plant growth and productivity [29,30,78]. As mentioned above, repression of the expression of *OsAAP5* or *OsAAP3* increases rice tiller number and grain number per plant, ultimately leading to a significant improvement in rice grain yield [29,78]. These valuable genes can be applied in rice breeding programs and may contribute to cultivating high-yield rice cultivars in the future. In Arabidopsis, loss-of-function of *AtAAP2* reduces the source-sink translocation of amino acids and thus more N is allocated to the mutant leaves. Increase in the leaf N supply leads to higher rubisco levels, chlorophyll content, photosynthetic rates and C assimilate export from leaves for sink C supply in *ataap2* plants. Consequently, the branch and silique number per plant are elevated to different extents in *ataap2* plants, ultimately resulting in an increase in total seed and oil yield [30,95]. This study indicates that decrease in source-to-sink transport of amino acids does not necessarily reduce seed yield, whereas it represents an alternative strategy to boost crop productivity.

### 5.2. Seed/Fruit Quality Improvement

About 65% of the global edible protein supplied for human nutrition is derived from plants [96]. Hence, the contents of protein and amino acids are key factors determining the nutritional quality of seeds. Generally, all proteinaceous amino acids are transported through phloem from source leaves to seeds, and then are used for synthesis of proteins. Altering amino acid transport via regulating transporters can ultimately affect the amino acid and protein content of seeds [26,97,98,99,100,101]. Rice is generally thought of as a cereal with lower grain protein content (GPC) [102], while significant genetic variations in GPC exist among different rice varieties [103]. Peng et al. [99] isolated a gene controlling GPC in the rice grain by a map-based cloning strategy, which encodes a putative amino acid transporter OsAAP6. Its transcripts are ubiquitously expressed in a variety of organs, but most abundant in the developing endosperms. When the coding region of *OsAAP6* from the high-GPC variety was overexpressed in the low-GPC variety, the engineered plants increase GPC. In contrast, RNAi-mediated knockdown of *OsAAP6* reduces GPC in the high-GPC variety [99]. Importantly, alteration in *OsAAP6* expression has no significant impact on other agronomic traits such as grain yield. Therefore, manipulating *OsAAP6* represents a valid strategy for enhancing GPC in rice and, potentially, other gramineous crops [99]. In the legumes pea and *Vicia narbonensis*, when the *Vicia faba* amino acid permease *VfAAP1* was specifically expressed in their embryos, transgenic seeds had higher amino acid import rates compared with the wild type, ultimately leading to elevated seed N and protein content [100].

In addition to increased amino acid and protein content in seeds, the palatability or quality of fruits can also be improved via genetic manipulation of amino acid transporters. The content of acidic metabolites is an important factor in determining tomato fruit quality. As the fruit ripens, the acidic amino acids aspartate and glutamate accumulate in the vacuole, while GABA level declines [104]. The tonoplast-localized transporter SlCAT9 was found to mediate the export of GABA from the vacuole in exchange for the import of aspartate or glutamate. Its overexpression resulted in many-fold increases in the contents of all three amino acids in ripe fruit [105]. Thus, amino acid transporters can also serve as valuable engineering targets for improving fruit flavor and nutritional quality.

### 5.3. Enhancing Plant Nitrogen Use Efficiency

In agricultural production, N fertilizers are usually applied to obtain a high yield, but crops take up only 30–40% of the applied N fertilizer [106]. The remaining soil N may be lost through surface runoff, leaching, denitrification and volatilization, which cause serious environmental pollution. To reduce production costs and potential environmental risks, increasing the nitrogen use efficiency (NUE) of crops is an urgent issue in agricultural production. NUE is defined as the total biomass or grain yield produced per unit of applied fertilizer N, which is generally composed of two physiological components: nitrogen uptake efficiency (NUpE) and nitrogen utilization efficiency (NUtE) [107]. NUpE is defined as the total N in plants relative to the applied N fertilizer, reflecting the capacity of plants to acquire N from the soil, while NUtE is defined as plant biomass or seed yield relative to total N in the plant, reflecting the capacity of plants to convert acquired N to plant biomass or seed yield [107]. Nowadays, many genes involved in inorganic N uptake, allocation and assimilation, as well as their regulation, have been used as genetic engineering targets for improving plant NUE [2,108]. However, taken into account that (a) as the main transport forms of organic N in most plants, large amounts of amino acids are required for transport via vasculature to the sink organs, supporting their growth, development and fruit/seed set, and (b) N is a highly mobile element. Proteins are degraded in older and senescing leaves, and then transported mainly in the form of amino acids and peptides to developing organs for reuse [109], manipulating amino acid transporters can also promote N allocation and reutilization within plants, eventually increasing NUE and seed yield.

Indeed, improved NUE has been reported in different plant species by altering the expression of amino acid transporters [78,95,110,111]. For example, pea plants overexpressing *PsAAP1* in the leaf phloem and embryos exhibited increased source-to-sink translocation of amino acids, seed yield, as well as NUE under low, moderate and high N environments [110]. *PsAAP1*-OE plants display improved NUtE under low and moderate N supply, while no difference in NUtE was found in a high N condition. Unlike NUtE, NUpE was unaltered in *PsAAP1* overexpressors under low N conditions but was enhanced when moderate or high N was supplied [110]. More importantly, *PsAAP1*-OE plants grown under moderate N condition produce similar yields to the wild type supplied with high N (twice the moderate N) [110]. These results indicate that modulating source-to-sink translocation of N could be a feasible strategy for improving crop NUE.

Different from *PsAAP1*, disruption of *AtAAP2* leads to enhanced NUE in Arabidopsis plants in a range of N environments [95]. Detailed analyses showed that the increase in NUE is caused by the higher NUpE in *ataap2* knockout plants, while the NUtE is unchanged [95]. The lack of difference in NUtE between *ataap2* and wild type is probably because loss of function of AtAAP2 reduces N transfer from vegetative organs to seeds, as indicated by observations that more N is retained in *ataap2* stubble tissues at harvest, while *ataap2* seed N content is reduced [95].

Unexpectedly, both PsAAP1 and AtAAP2 are involved in transport and partitioning of amino acids in the shoot, but alterations in their expression simultaneously enhance root N uptake and assimilation, as well as root-to-shoot N delivery [94,95,110]. The reasons for this effect are very complex. First, changes in shoot N concentrations or status probably influence shoot-to-root signaling and trigger positive feedback regulation of root N uptake and assimilation [94,95]. Alternatively, alterations in shoot N metabolism affect plant C metabolism (probably increased photosynthesis and sugar delivery), and C assimilates provide the energy and C skeleton for root N uptake and assimilation [94,95]. 

It should be noted that the increased plant yield and NUE presented in these studies is obtained usually from pot experiments and under controllable environments. Whether the good performances of these genetically modified plants can be achieved in farmland is still to be tested. But anyway, altering plant N transport and allocation by manipulating amino acid transporters presents a highly promising strategy for increasing crop yield and NUE.

## 6. Concluding Remarks

Dedicated transport proteins are required to mediate intracellular and intercellular translocation, and long-distance transport of amino acids within plants. With the completion of genome sequencing of different plant species, a large number of amino acid transporters have been annotated in plants. Recently, considerable progress has been made in functionally identifying plant amino acid transporters. Due to their broad expression pattern and substrate specificity, these transporters may have more than one physiological function in planta, which requires further exploration. In addition, the functional characterization of plant amino acid transporters is mainly performed in the model plant Arabidopsis, while research on their counterparts in major crops is still scarce, which also needs to be investigated in the future. Furthermore, genetic manipulation of some amino acid transporters can enhance plant biomass, seed yield and/or quality, as well as NUE, which offers a promising strategy for crop improvement.

## Figures and Tables

**Table 1:** Number of amino acid transporter family (ATF) and amino acid-polyamine-choline transporter (APC) family members in some plant species.

Species	Total	ATF/AAAP ^a^								APC			Others	References
		AAP	LHT	ProT	GAT	ANT	AUX	ATL ^b^	VAAT ^b^	CAT	ACT	PHS	TTP ^c^	
*A. thaliana*	63	8	10	3	2	4	4	5	10	9	1	5	2	[17,18]
rice	85	19	6	3	4	4	5	7	10	11	7	9	N. D. ^d^	[19]
soybean	189	35	24	7	19	6	16	16	30	19	7	9	1	[18]
poplar	100	17	13	3	7	4	8	8	11	16	6	7	N. D.	[20]
maize	96	24	15	2	2	3	5	6	14	12	7	6	N. D.	[20,21]
potato	72	8	11	4	3	5	5	8	8	9	1	8	2	[9]
wheat	283	60	24	9	12	18	15	55 ^e^		31	19	31	9	[22]
*R. communis*	62	10	9	7	N. D.	15 ^f^	4			15	2	N. D.	N. D.	[23]
*M. truncatula*	86	26	18	3	4	3	5	13	14	N. D.	N. D.	N. D.	N. D.	[24]
*P. edulis*	55	16	8	3	6	2	7	6	7	N. D.	N. D.	N. D.	N. D.	[25]

^a^: The full names of the abbreviations, ATF, APC, AAP, LHT, ProT, GAT, ANT, AUX, ATL, VAAT, CAT, ACT and PHS, can be found in the main text. AAAP: amino acid/auxin permease; TTP: tyrosine-specific transporter; *A. thaliana*: *Arabidopsis thaliana*; *R. communis*: *Ricinus communis*; *M*. *truncatula*: *Medicago truncatula*; *P. edulis*: *Phyllostachys edulis*. ^b^: The ATL and VAAT subfamilies are also known as ATLa and ATLb, respectively. ^c^: TTP, tyrosine-specific transporter, is classified in APC family in reference [9] but is classified in ATF family in reference [22]. ^d^: N.D. not determined. ^e^: This contains the members of VAAT subfamily. ^f^: This contains the members of ATL and VAAT subfamily.
